# Learning intraprofessional collaboration by participating in a consultation programme: what and how did primary and secondary care trainees learn?

**DOI:** 10.1186/s12909-017-0961-9

**Published:** 2017-07-19

**Authors:** Marijn Janssen, Margaretha H. Sagasser, Elisabeth A. M. Laro, Jacqueline de Graaf, Nynke D. Scherpbier-de Haan

**Affiliations:** 1 Department of Internal Medicine, Radboud University Medical Centre, Post box 9101, 6500 HB Nijmegen, the Netherlands; 2 Department of Primary and Community care, Radboud University Medical Centre, Post box 9101, 6500 HB Nijmegen, Nijmegen, the Netherlands; 3 National postgraduate training programme in general practice, Post box 20072, 3502 LB, Utrecht, the Netherlands

**Keywords:** Intraprofessional collaboration, Postgraduate education, Learning outcomes, Qualitative method, Focus groups, Internal medicine, General practice

## Abstract

**Background:**

A growing number of patients require overview and management in both primary and secondary care. This situation requires that primary and secondary care professionals have well developed collaborative skills. While knowledge about *inter*professional collaboration and education is rising, little is known about *intra*professional collaboration and education between physicians of various disciplines. This study examines a newly developed consultation programme for trainees in general practice and internal medicine to acquire intraprofessional collaboration skills.

**Methods:**

Focus groups were conducted with trainees and their supervisors and mentors to explore what and how the trainees learned by participating in the consultation programme.

**Results:**

Trainees reported that they gained knowledge about and skills in collaboration and consultation they could not have gained otherwise. Furthermore, the programme gave the opportunity to gain other competencies relevant for becoming the medical expert trainees they are expected to be. Learning outcomes were comparable to those described in interprofessional education literature. Interaction, by meeting each other and by discussing cases with mentors or supervisors, appeared to be a key factor in the learning process. Meetings, discussing preconceptions and enthusiasm of the mentors and supervisors facilitated the learning. Technical problems and lack of information hampered the learning. These influencing factors are important for future development of intraprofessional learning programmes.

**Conclusions:**

Participants in an innovative consultation programme for GP- and IM-trainees reported that they acquired consultation and collaboration skills they could not have gained otherwise. Interaction appeared to be an important factor in the learning process. The findings of this study can inform developers of intraprofessional education programmes between primary and secondary care trainees.

**Electronic supplementary material:**

The online version of this article (doi:10.1186/s12909-017-0961-9) contains supplementary material, which is available to authorized users.

## Background

Medical specialty training in the Netherlands, as in many other countries, is competency based. The CanMEDS framework is used as the basic framework in the different training programmes [1]. One of the key CanMEDS roles of the medical expert is that of Collaborator. CanMEDS describes three core competencies for this collaborator role: work effectively with physicians and other colleagues in the health care professions; work with physicians and other colleagues in the health care professions to promote understanding, manage differences, and resolve conflicts; and hand over the care of a patient to another health care professional to facilitate continuity of safe patient care. Education in collaboration is important to provide medical specialty trainees with the described collaborative competencies.

**Fig. 1:**
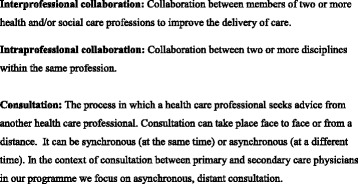
Explanation of the definitions inter- and intraprofessional collaboration and consultation [3, 11]

*Inter*professional collaboration and education (see Fig. 1) are widely described in literature as key factors in increasing the effectiveness of health services. Interprofessional education has proven to have a positive effect on knowledge about, attitudes towards and behaviour in interprofessional collaboration; and on organizational and patients outcomes [2–4].

*Intra*professional collaboration and education between primary and secondary care physicians is less well studied, but becomes more and more important due to demographic shifts and advancing technical and medical possibilities. Postgraduate training programmes, however, do not provide formal training in this intraprofessional collaboration and studies on this subject are scarce [5, 6]. In one of the few studies published on this topic, trainees reported that intraprofessional collaboration led to a better understanding of one another’s professional roles, responsibilities and behaviour. They observed that most intraprofessional learning takes place informally, in the context of patient care [7].

Literature describes barriers for intraprofessional collaboration at the primary secondary care interface that could be overcome with intraprofessional education. These barriers are: not knowing one another; impaired knowledge about each other’s working space; unclear roles and responsibilities; lack of mutual respect; and questioned expertise [6, 8, 9]. These barriers probably develop during postgraduate education, since this is the first time physicians work and learn in separate contexts, and do not share a common education programme.

To explore the development of intraprofessional collaboration skills during postgraduate education, we designed an innovative consultation programme for primary and secondary care trainees. The programme focuses on the consultation process because collaboration between physicians of primary and secondary care often takes place in the form of consultations and work related activities play a central role in the learning of trainees [10].

The consultation programme was offered to general practice trainees (GP-trainees) and internal medicine trainees (IM-trainees). The programme aimed to create the opportunity to learn intraprofessional collaboration through consultation between the trainees of the two disciplines. We conducted a qualitative study to gain deeper insight into what participants had learned, and what processes and activities contributed to their learning. The aim of this study was to elicit what and how GP- and IM-trainees learned from their participation in the consultation programme. The results can be used to further develop intraprofessional education programs for primary and secondary care trainees.

## Methods

### Research context

#### Postgraduate training

This research was conducted at the Radboud University Medical Centre (Radboudumc) in the Netherlands. It was performed within the context of the three-year postgraduate training for general practice (GP-specialty training) and the six-year specialty training for internal medicine in the Netherlands. In year one and three of the GP-specialty training, GP-trainees work in general practice where they are coached and instructed by one supervisor (GP-supervisor). The trainees work in general practice for four days per week and on the fifth day they attend a day-release programme in groups of approximately ten trainees facilitated by two mentors (GP-mentors). During the second year, trainees complete rotations in emergency rooms, nursing homes and psychiatric outpatient clinics with different supervisors.

The six-year training for internal medicine takes place in academic and non-academic hospitals. In the first year trainees are trained in general internal medicine, mostly in regional hospitals. During the following three years trainees take rotations in which they work in various departments such as the outpatient clinic and the ICU in either a regional or academic hospital. In the last two years trainees choose one or a combination of two or three subspecialisations such as oncology or nephrology. They receive daily supervision from a supervisor (IM-supervisor) [5].

#### The consultation programme

Work related activities play a central role in the learning of trainees during their postgraduate training. An important aspect of collaboration between primary and secondary care physicians are the processes of referral and consultation. We chose to focus on the process of consultation as a means of learning and promoting intraprofessional collaboration.

**Fig. 2:**
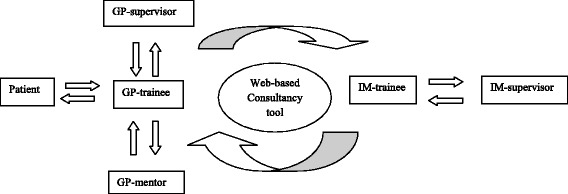
The consultation programme (GP = general practice, IM = internal medicine)

The consultation programme (Fig. 2) had two goals. Firstly to learn how to ask for, and how to provide consultation advice. Trainees had to learn to briefly and correctly formulate consultative questions and answers. Secondly, the programme aimed to explore educational activities to promote intraprofessional collaboration.

The programme started in November 2014. GP-trainees were in the third year of their training, the IM-trainees in their fourth, fifth or sixth year. Participation was voluntary. In total forty GP-trainees and their eight mentors, and twelve IM-trainees and their three supervisors participated in the programme. Due to scheduling of their individual training programme GP-trainees could enrol or exit during the course of the programme. Every four months another group of four IM-trainees participated in the programme.

#### The consultation process and supervision

In this innovative consultation programme GP-trainees were offered the opportunity to consult IM-trainees about patients with a problem in the field of internal medicine, where they felt uncertainties. This communication took place through a web-based tool that was linked to the Electronic Patient Record (EPR) in general practice. The GP-trainees could extract relevant data such as medical history, medication and laboratory results from the EPR and automatically insert these in a web-based consultation form. IM-trainees were informed by mail when a new consultation arrived. They had 48 h to answer the question. Their advice was electronically sent to the GP-trainee and imported in the EPR. IM-trainees had a biweekly meeting with their supervisor to discuss their answers to the consultations.

#### Intraprofessional meetings

At the start of the programme GP-trainees, IM-trainees, IM-supervisors and GP-mentors were invited to a kick-off meeting: a plenary session to become acquainted with the goals and the working procedures of the programme and to meet each other. The two different training programmes were discussed and trainees were challenged to discuss expectations of and preconceptions about one another. After seven months a lunch meeting was organized so that GP-trainees and IM-trainees could exchange experiences and discuss examples of consultations. GP-trainees and IM-trainees were encouraged to invite each other to visit their workplace in order to gain a better understanding of each other’s workplaces.

### Research design

To explore and explain what and how the trainees learned from the consultation programme we conducted a qualitative study [12]. We chose focus group research because interaction between group members can lead to an in-depth discussion of topics [13].

### Data collection

Three focus groups were organised with 1) GP-trainees, 2) IM-trainees and 3) GP-mentors and IM-supervisors. We chose a homogeneous composition of the groups, so that participants would feel safe to express conflicts or concerns [13]. Due to the small number of available GP-mentors (eight) and IM-supervisors (four) a combined focus group was composed including these two groups. We aimed to include six to ten participants in each focus group. Trainees had to have participated in the programme for at least three months.

All eligible participating IM-trainees, GP-mentors and IM-supervisors were approached by e-mail. GP-mentors were asked to approach all eligible GP-trainees during their day release programme. The focus groups took place at the Radboud university medical centre, seven months after starting the consultation programme. At that moment two groups of IM-trainees (eight trainees) had taken part in the programme and approximately forty GP-trainees had had the possibility to make a consultation. Before participating in the focus groups, the participants had to give informed consent.

One female interviewer (MS), an educationalist with experience in conducting focus groups, guided the focus group interviews and a research assistant (EL) took additional notes. Interview topics were derived from the research questions. An interview topic list was used (Additional files 1, 13]. The interviewer made sure all topics were discussed. Emerging questions from a focus group were discussed in subsequent focus groups.

### Data analysis

The focus group interviews were audiotaped and transcribed verbatim and anonymised. The transcripts were analysed using qualitative content analysis [14]. Transcripts were read and reread, relevant text fragments were coded by two researchers (MS and EL) followed by identifying themes and patterns. These results were discussed in the research team (MS, EL, MJ, NS, JG), resulting in description of findings. Differences in coding between the two coding researchers were solved by discussion. Atlas.ti7 version 7.1.5. was used to organize the data. A deductive and an inductive approach to analysis were combined, as this allows for using predefined topics as well as topics emerging from the data. Data analysis started as soon as the first data was gathered. So, data gathering and data analysis were iterative processes, thereby promoting dependability [12, 15]. During analysis, subthemes, themes and topics were derived. By frequently discussing the analytic process and the findings in the research team, reflexivity and thereby confirmability was achieved [12, 15, 16]. To increase credibility, member checking was applied by sending participants a summary of their focus group interview, giving them the opportunity to review the researchers’ first interpretation [15, 17]. Further triangulation was achieved by discussing emerging questions from one group in the following focus groups [15, 17].

## Results

Eighteen participants participated in three focus groups (Table 1). In general, the participating trainees and their mentors and supervisors were enthusiastic about the consultation programme. This enthusiasm was important to encourage the trainees to make more consultations and thereby increase the learning effect. We will first present the results on *what* trainees learned and *how* they learned, and subsequently factors that influenced the consultation and learning of the trainees will be presented. An additional file shows an example of the process of coding and abstraction of themes and subthemes (additional file 2).

**Table 1:** Characteristics of the focus groups

	GP-trainees	GP-Mentors & IM-Supervisors		IM-trainees
Number of participants	7	3	3	5
Gender				
Male	2	1	1	1
Female	5	2	2	4
Duration focus group	57 mins	63 mins		47 mins
Order	1	2		3

*GP* general practice, *IM* internal medicine

### What did the trainees learn

We identified five themes: collaboration, consultation, knowledge acquisition, professionalism and health advocacy (Table 2).

**Table 2:** Results on what trainees learned

	FG GP trainees	FG IM trainees	FG Mentors & Supervisors
Collaboration Knowledge about the working environment	• What happens with the patient after referral to IM • Knowing what an IM-trainee does	• GP sees a different population • What diagnostic tests are possible in a GP-practice • In the hospital everything is easily arranged but for a GP that is different • GP has a different way of working and sees the patient in a different way • Put yourself in position of the GP • GP has to make a difficult decision: referral or not?	• IM-trainees are more aware of what happens before referral to IM • Both trainees learned what each other’s training looks like
Knowledge about the medical knowledge of the other professional		• IM-trainees learn a lot that GP-trainees do not learn • Maybe GP-trainees do not ask themselves why they do certain blood tests and maybe they do not know what blood tests are clinically relevant to send along with the consultation (since a lot of consultations were about blood tests) Maybe GP-trainees find it hard to identify topics for consultations. Maybe that is why they bring questions that you can look up in literature • IM-trainees might know better where to search for information in literature	
Collaborative attitude	• The approach was pleasant • GP-trainees are taken seriously	• Challenge to see the patient through the eyes of the GP	• For GP-trainees, the programme promotes the approachability of the specialist in the hospital • GP-trainees feel that they are taken seriously • IM-trainees take the programme seriously
Consultation	• To formulate a good question • To give a clear summary of the case	To formulate a good answer Uncertainty about written communication: is it clear? Does it come across as patronising?	• GP-trainees learned to formulate a good question • IM-trainees learned to formulate a good answer • IM-trainees are insecure about sending an explanation with the consultation answer: “Can I just do that?” • IM-trainees do not want to come across as being pedantic
Knowledge acquisition	• Gain knowledge for the next patient	Gain knowledge beyond own subspecialty in IM	• GP-trainees acquired medical knowledge in general • For IM-trainees it’s good to consider that you can do a lot without blood tests
Professionalism	• Be more involved with the patient or in patient care • Develop a critical attitude: what do I look up myself? What will I do with the answer of the IM-trainee? • Take responsibility for own consultation	Be more critical towards GP-trainee (e.g.: ask why GP-trainee did certain blood tests)	• For GP-trainees, the programme promotes an investigative approach • IM-trainees learned to be independent and take responsibility
Health advocacy	• Programme could possibly bridge the gap between primary and secondary care • Nice intervention for doubtful cases • Patient receives customized advice • Patient gets to hear what happens after referral to the hospital • Patients receive secondary intervention at the GP practice and do not need to arrange transport to the hospital • This programme could possibly reduce the amount of referrals required • A higher level of health care in the GP practice is cheaper		• Programme reduces the amount of referrals required

*FG* focus group, *IM* internal medicine, *GP* general practice

#### Collaboration

Knowledge of the other’s working environment and medical expertise were important items that were mentioned frequently during all focus groups. IM-trainees in particular learned a lot on this subject.

IM-trainee: “What I did learn is that a GP sees a different population than we do and also has another way of working so you have to put yourself in position of the GP.”

With regards to a collaborative attitude, GP-trainees felt they were taken seriously by IM-trainees. GP-mentors and IM-supervisors found it important that the trainees took each other seriously. In the following quote GP-mentors and IM-supervisors exchange experiences about this subject:

IM-supervisor: “It was just taken very seriously, I noticed that they (IM-trainees) examined and answered every question very seriously indeed …”

GP-mentor: “Yes, because when I think about it too, apparently this can have as result that GP-trainees feel taken seriously as a GP,... say “Right, because I receive such extensive advice I am taken seriously .””

#### Consultation

In all focus groups the formulation of consultation questions and answers was a recurrent subject. The programme offered learning opportunities in this field, which they did not experience in the rest of their training programmes. GP-trainees learned to formulate a good question. But they mentioned that they felt insecure to ask advice on issues which they perceived to be “simple problems”. IM-trainees learned to formulate a good answer but were sometimes a bit insecure about their formulations, because they did not want to come across as patronizing.

#### Knowledge acquisition

IM-trainees found it useful to learn information that extended beyond their IM-subspecialisation. GP-trainees reported the consultations provided knowledge that could assist in the treatment of future patients.

GP-trainee: “... with a minimal anaemia you think, well, generally speaking you would wait and see. But when you can ask such a question to the internist and you know what analyses you could do in that case, then you know that for your next patient too.”

#### Professionalism

GP-trainees learned to take responsibility for the consultation and became critical towards themselves.

GP-trainee: “… you have to think carefully about the question you’re about to ask. So you have to be critical towards yourself like “What can I look up myself?”… And also think about what you’re going to do with the answer.”

GP-mentors mentioned that the programme promotes an investigative attitude. IM-trainees learned to be more confident in their answers towards GP-trainees. IM-supervisors reported different learning points on this theme such as independence and taking responsibility.

Interviewer: “Can you report what you noticed that the trainees have learned?” IM-supervisor: “Independence; that they dare to answer, right away, because they actually do that without previously conferring with us.”

#### Health advocacy

GP-trainees mentioned many learning points on health advocacy, for example the fact that consultation could save referrals and possibly bridge the gap between primary and secondary care.

GP-trainee: “Well, I found the treatment and the collaboration just very pleasant. Yes I think that really stimulates to collaborate more and to bridge the gap between primary and secondary care. I think that’s good for the future.”

GP-trainees reported that they felt the programme had some advantages for the patient, like receiving essential care in their own neighbourhood if possible*.*

GP-trainee: “Yes well maybe the patient is a stimulating factor, because the patient generally wants to get his health care at his own GP-practice. And if the same care is offered both at his own GP-practice and at the hospital, he will often choose for his own GP-practice. … And they don’t have to arrange transport to the hospital.”

### How did the trainees learn

Trainees reported that the programme encouraged them to acquire knowledge that they would not have acquired otherwise. The ways in which trainees learned can be divided in individual learning and learning by interaction.

#### Individual learning

Both trainee groups were encouraged to look up medical knowledge. GP-trainees sought information from literature before the consultation with the aim to formulate a focused question and after the consultation to gain more knowledge on the subjects addressed by the IM-trainees.

IM-trainees sought information from literature to formulate a good answer and gained insight into the guidelines of the GPs. Trainees gained individual skills like formulating questions and answers by engaging in consultation.

#### Learning by interaction

Interaction, with other trainees and with supervisors, was identified as a key-factor in the learning process. GP-trainees and IM-trainees could mail back and forth which they found beneficial for their learning. Mailing IM-trainees offered GP-trainees the opportunity to ask additional questions. Furthermore they learned about each other’s working environment. IM-trainees would have liked more feedback on their answers, so that they could learn if the advice they gave was useful.

IM-trainee: “So, if they give feedback on our answers, if it’s too large or too easy or too complicated. Then you can learn something.”

Learning by interaction also occurred during meetings. All participants found these meetings informative and encouraging. Mentors and supervisors noticed that through the kick-off meeting, trainees got to know the essentials of the training of the other. By discussing prior consultations at the lunch meeting they learned what types of questions were suitable for consultation.

IM-trainees found the biweekly meetings in which they reflected on their answers with the IM-supervisor informative. IM-supervisors gave tips about the content and the form of the advice the IM-trainees gave.

IM-supervisor “And I also tried to encourage them not to confine themselves with an answer but to also add an explanation like “Well you could act a bit like you’re teaching so also explain why or what you would also like to know and why and what thoughts lie behind that” and yes they picked that up.”

### Factors influencing intraprofessional learning

Besides the answers to our research questions several factors influencing the consulting and thereby the learning of the trainees were identified. The factors associated with trainees, with supervisors and mentors and with the learning context will be presented. Influencing factors can function as facilitators or barriers. These factors are important for the future development of intraprofessional learning programmes between primary and secondary care physicians. The facilitating factors include success factors. The barriers highlight challenges which have to be taken into account during the development and implementation of future programmes.

#### Factors associated with the trainees

The trainees were largely of the same age and in the same position. As a result they experienced little hierarchy and a low threshold to get in touch with each other.

GP-trainee: “Well it’s just very easy to approach each other because you’re both about the same age ... instead of, well, the hospital, the sacred hospital, it’s now more like you’re on the same level.”

GP-mentors saw that GP-trainees with an investigative nature were more likely to consult. GP-trainees with high self-perceived knowledge on internal medicine felt less need to consult an IM-trainee.

#### Factors associated with the supervisors and mentors

Enthusiasm of the GP-mentors and supervisors stimulated trainees to consult. GP-trainees found that GP-supervisors and mentors with a negative disposition and GP-supervisors with high self-perceived knowledge on internal medicine, discouraged them to make a consultation. When GP-mentors and GP-supervisors paid little attention to the programme, the number of consultations decreased.

#### Factors associated with the context

The consultation programme took place in the workplace, and the information gathered by the GP-trainees from making a consultation was directly applicable in patient care. The care needs of the patient functioned as a stimulator to consult.

Facilitating meetings for trainees, mentors and supervisors was important to keep the consultation going. During this meetings, consultation questions were discussed leading to ideas for new consultations. Trainees were able to learn about each other’s workplace, roles, responsibilities and possibilities. Trainees talked about the expectations they had of the programme and of each other. During the kick-off meeting they exchanged stereotypes of each other, making consulting one another easier.

IM trainee: “And then we got to talk about the preconceptions of one another and yes, that you can hear what we think of one another and that also opens doors. I found that very nice.”

Technical problems, like problems with the computer system and the log on codes, were barriers to start a consultation.

## Discussion

The aim of this study was to elicit what and how GP- and IM-trainees learned from their participation in the consultation programme. Analysis revealed five themes: Collaboration, Consultation, Knowledge Acquisition, Health Advocacy, and Professionalism. Trainees reported that they gained knowledge about collaboration and consultation they could not have gained otherwise. Furthermore, the programme gave the opportunity to gain other knowledge and skills relevant for becoming a medical expert, like medical knowledge, health advocacy and professionalism [1]. Interaction with supervisors, mentors and other trainees appeared to be of high importance in the learning process.

The programme focused on consultation. In this process the GP-trainees are expected to take an active role in asking for information and IM-trainees a responsive role in providing information. Despite the responsive role of the IM-trainees the programme offered learning experiences about consultation and medical knowledge for both IM- and GP-trainees. The mailing and meetings added knowledge for both disciplines about the different work environments, diagnostic and therapeutic possibilities and guidelines. This extended learning effect can help to promote mutual respect and understanding of different roles and responsibilities within the different disciplines.

Even though the GP-trainees experienced working in the hospital before, they reported gaining new knowledge and skills about intraprofessional collaboration. This may be explained by the fact that consultation is a specific action they do not learn during their rotations. Also, when they take their rotations in the various disciplines, their learning is focused on medical knowledge in the specific fields and not on (future) collaboration.

In one of the scarce studies on intraprofessional collaboration, Sibert et al. used focus groups to determine essential skills for good communication between consultants (urologists) and referring physicians. They identified two groups of skills: “observable skills” and “principles and attitude”. Skills they identified comparable to our findings are communication, formulating a good question and a clear answer, knowledge on expertise of the referring physician and mutual respect. Sibert et al. emphasized the importance of the determination of roles and responsibilities [18]. In our study, roles and responsibilities were well defined as part of the programme: the GP-trainee continued to be the patients’ main caregiver who was responsible for the further treatment of the patient and had to decide to act upon the advice or not. IM-trainees were responsible for their advice.

More research has been conducted and published in the field of interprofessional collaboration. Comparing our learning outcomes to those of a review on interprofessional education by Thistlethwaite and Moran reveals many similarities. Thistlethwaite and Moran reported in their qualitative synthesis of literature on learning outcomes of interprofessional education. They found six themes: Teamwork, Roles/Responsibilities, Communication, Learning/Reflection, the Patient, and Ethics/Attitudes [19]. The themes in our research, namely, Collaboration, Professionalism, Consultation, Knowledge acquisition and Health advocacy are comparable to the first five themes. We found that mutual respect and discussing stereotypes (Ethics/Attitudes) were important factors to promote consultation and learning.

Even though primary and secondary care physicians have the same profession and underwent the same graduate education, their postgraduate training programmes are separated and take place in different contexts. In today’s postgraduate training programmes little attention is paid to the acquisition of collaborative competencies between primary and secondary care physicians. It is possible that *intra*professional collaboration and education between primary and secondary care doctors face the same challenges as *inter*professional collaboration and education.

A strength of our research is that it is aimed at intraprofessional education and collaboration. To our knowledge this is one of the few studies focusing on intraprofessional education between physicians from primary and secondary care. Another strength is the multiple perspectives that are included in this research: trainees and supervisors from both primary and secondary care involved in the programme were able to share their opinions and experiences in the focus groups. Furthermore, the focus group design suited the research questions well. By interaction during the focus groups a deeper insight into the learning processes was gained.

At the time of the focus group it turned out that two participants in the GP-trainees focus group had just enrolled in the programme and therefore did not meet the inclusion criteria. Thus learning outcomes are based on the experiences of five GP-trainees, a small number comparing to the number of GP-trainees in the programme. However, the aim of qualitative research is to obtain a rich description of the subject studied and sample size is determined by data saturation [17]. In the third focus group GP-mentors, who spoke to several GP-trainees on a weekly basis, reported similar learning outcomes as the GP-trainees. We therefore judged that data saturation was reached. The input of the two trainees that just enrolled the programme was useful to identify influencing factors on their learning, like enthusiasm of the mentors and supervisors as facilitators and technical problems and lack of information as barriers to initiate consultations. It is also important to bear in mind a possible selection bias in the results. All participants participated voluntarily in the programme and the focus groups. It is possible that the participants of the focus groups were more eager to learn during the programme and to share their opinions than those who chose not to participate.

Overall, this study is innovative in exploring possibilities for intraprofessional learning of primary and secondary care physicians in the postgraduate training environment. In our study the fact that the learning took place in the work context, knowledge could be directly transferred to patient care and meeting each other were elements of the intervention that facilitated the learning. There is a definite need for further research on interventions in the field of intraprofessional education to determine the nature of effective educational interventions, desired learning outcomes and their measurement. It is important to find out what works, for whom, and why in order to develop educational interventions that are both effective and efficient considering the already full training programmes. There are signs that intra- and interprofessional collaboration and education have several similarities, so lessons from the increasing amount of literature in interprofessional education can inform research about intraprofessional education. With this research we could be moving a step closer to training professionals’ intraprofessional collaborative competencies in order to optimize patient centered health care.

## Conclusions

Participants in an innovative consultation programme for GP- and IM-trainees reported that they acquired consultation and collaboration skills they could not have gained otherwise. Interaction appeared to be an important factor in the learning process. The findings of this study can be helpful in the development of other intraprofessional education programmes between primary and secondary care trainees.

## Additional files

Additional file 1: Interview questions for the focus groups. (DOCX 13 kb)

Additional file 2: Example of the process of coding and abstraction of themes and subthemes. (PDF 443 kb)

## Abbreviations

FG: Focus group
GP: General practice
IM: Internal medicine

## References

[1] The Royal College of Physicians and Surgeons of Canada. http://www.royalcollege.ca/rcsite/canmeds-e. Accessed Apr 2017.

[2] Reeves S, Perrier L, Goldman J, Freeth D, Zwarenstein M (2013). Interprofessional education: effects on professional practice and healthcare outcomes (update). The Cochrane database of systematic reviews.

[3] Gilbert JH, Yan J, Hoffman SJ (2010). A WHO report: framework for action on interprofessional education and collaborative practice. J Allied Health.

[4] Frenk J, Chen L, Bhutta ZA, Cohen J, Crisp N, Evans T (2010). Health professionals for a new century: transforming education to strengthen health systems in an interdependent world. Lancet (London, England).

[5] Koninklijke Nederlandsche Maatschappij tot bevordering der Geneeskunst. https://www.knmg.nl. Accessed June 2016. 13013448

[6] Beaulieu MD, Samson L, Rocher G, Rioux M, Boucher L, Del Grande C (2009). Investigating the barriers to teaching family physicians' and specialists' collaboration in the training environment: a qualitative study. BMC Medical Education.

[7] Meijer LJ, de Groot E, Blaauw-Westerlaken M, Damoiseaux RA (2016). Intraprofessional collaboration and learning between specialists and general practitioners during postgraduate training: a qualitative study. BMC Health Serv Res.

[8] Manca D, Varnhagen S, Brett-MacLean P, Allan GM, Szafran O. Respect from specialists: concerns of family physicians. Can family physician Medecin de famille canadien. 2008;54(10):1434–1435, 5.e1–5. PMC2567269 18854474

[9] Manca DP, Varnhagen S, Brett-MacLean P, Allan GM, Szafran O, Ausford A (2007). Rewards and challenges of family practice: web-based survey using the Delphi method. Can family physician Medecin de famille canadien.

[10] Teunissen PW, Boor K, Scherpbier AJ, van der Vleuten CP, van Diemen-Steenvoorde JA, van Luijk SJ (2007). Attending doctors' perspectives on how residents learn. Med Educ.

[11] Bainbridge LNL. Inter and intra-professional collaborative patient-Centred Care in Postgraduate Medical Education. Members of the FMEC PG consortium. 2011;

[12] Tavakol M, Sandars J (2014). Quantitative and qualitative methods in medical education research: AMEE guide no 90: part I. Med Teach..

[13] Stalmeijer RE, McNaughton N, Van Mook WN (2014). Using focus groups in medical education research: AMEE guide no. 91. Med Teach..

[14] Hsieh HF, Shannon SE (2005). Three approaches to qualitative content analysis. Qual Health Res.

[15] Frambach JM, van der Vleuten CP, Durning SJ (2013). AM last page. Quality criteria in qualitative and quantitative research. Acad Med.

[16] Barry CA, Britten N, Barber N, Bradley C, Stevenson F (1999). Using reflexivity to optimize teamwork in qualitative research. Qual Health Res.

[17] Tavakol M, Sandars J (2014). Quantitative and qualitative methods in medical education research: AMEE guide no 90: part II. Med Teach.

[18] Sibert L, Lachkar A, Grise P, Charlin B, Lechevallier J, Weber J (2002). Communication between consultants and referring physicians: a qualitative study to define learning and assessment objectives in a specialty residency program. Teaching and Learning in Medicine.

[19] Thistlethwaite J, Moran M (2010). World Health Organization study group on Interprofessional E, collaborative P. Learning outcomes for interprofessional education (IPE): literature review and synthesis. J Interprof Care.

