# Supplementary material for: Learning intraprofessional collaboration by participating in a consultation programme: what and how did primary and secondary care trainees learn?
Source: BMC Med Educ. 2017 Jul 19;17:125. doi: 10.1186/s12909-017-0961-9 (PMC5517789; doi:10.1186/s12909-017-0961-9)
Supplement: Supplementary file 1 — Interview questions for the focus groups. (DOCX 13 kb) [file 12909_2017_961_MOESM1_ESM.docx]

**Additional file 1: Interview questions**

**Focus group GP-trainees**

Opening question: Can you tell us who you are (name) and shortly describe what your best and worst experience was during the consultation programme.

1. Can you indicate what you learned about the collaboration between primary and secondary care?

2. Can you indicate what you learned about consulting (formulating the questions)?

3. Can you explain what helped you to learn that?

4. Can you explain what hindered you in learning that?

5. How did the patient experience the consultations?

6. Do you have tips and tricks which you could share?

7. If you could talk about the programme with the programme directors for one minute, what would you say?

8. Do you have any additional comments?

**Focus group GP-mentors and IM-supervisors**

Opening question: Can you tell us who you are (name) and shortly describe what your activities were within the programme? What was your role as GP-mentor / IM-supervisor?

1. What do you think that the GP-trainees and IM-trainees learned from the programme?

2. Can you describe what you learned of the programme yourself?

3. What do you think about the implementation and the progression of the programme?

4. Can you explain what contributed to the learning process of trainees?

5. Can you explain what hindered the learning process of trainees?

6. Do you have ideas for improvement for this programme which you could share?

7. Are there other positive outcomes of the programme that you are aware of?

8. Do you have any additional comments?

**Focus group IM-trainees**

Opening question: Can you tell who you are (name) and in what period you answered questions in the consultation programme?

1. Can you describe how the process of answering the questions takes place?

2. Can you indicate what you learned about collaboration between primary and secondary care?

3. Can you indicate what you learned about consulting (answering the questions)?

4. What did you think of the questions of the GP-trainees?

5. How did you look at the collaboration?

6. Can you explain what helped you to learn that?

7. Can you explain what hindered you in learning that?

8. Do you have tips and tricks which you could share?

9. Do you have any additional comments?
