# Supplementary figures and images for: Learning intraprofessional collaboration by participating in a consultation programme: what and how did primary and secondary care trainees learn?
Source: BMC Med Educ. 2017 Jul 19;17:125. doi: 10.1186/s12909-017-0961-9 (PMC5517789; doi:10.1186/s12909-017-0961-9)

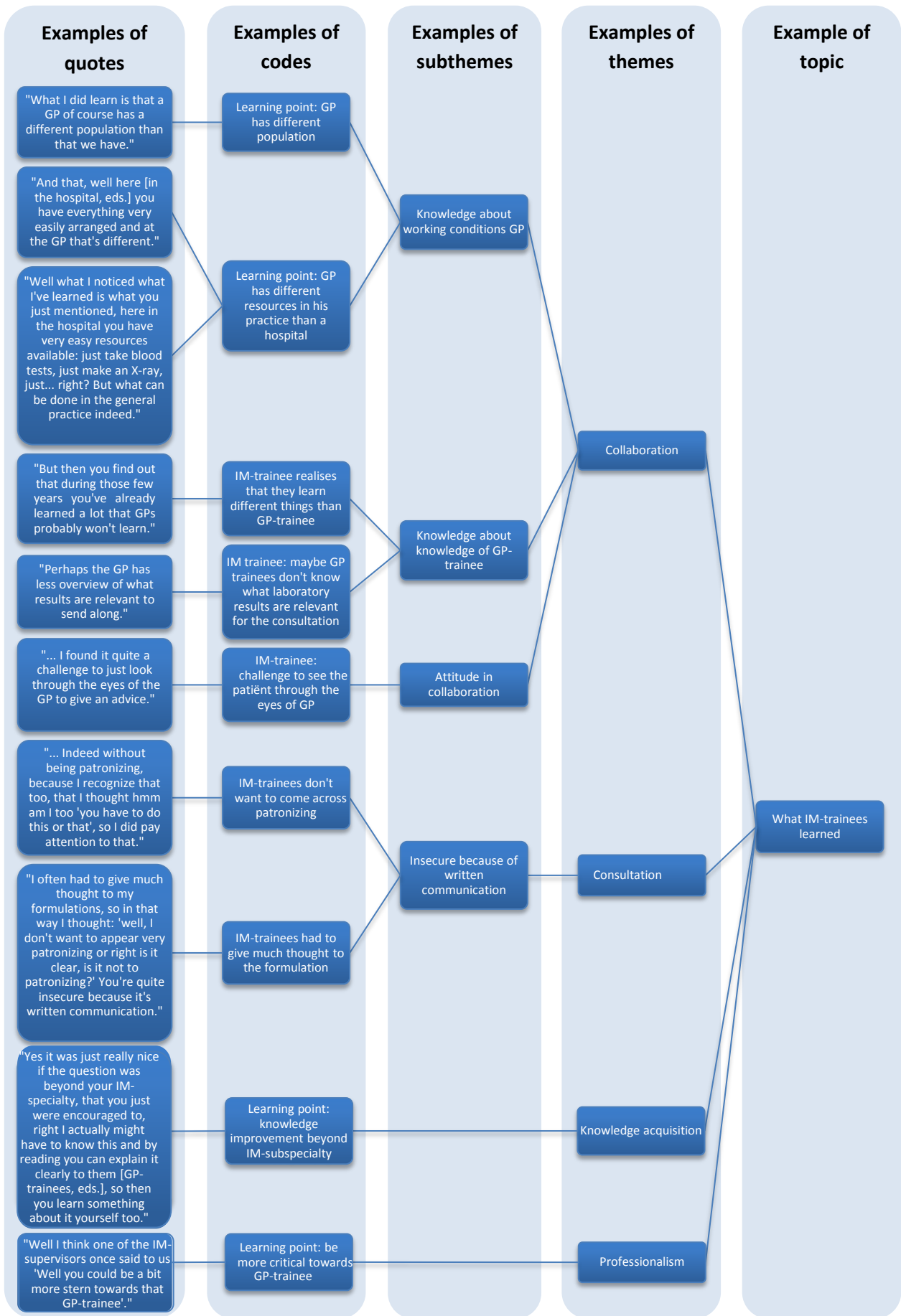

Supplement: Supplementary file 2 — Example of the process of coding and abstraction of themes and subthemes. (PDF 443 kb) [file 12909_2017_961_MOESM2_ESM.pdf]
